# Rs205764 and rs547311 in linc00513 may influence treatment responses in multiple sclerosis patients: A pharmacogenomics Egyptian study

**DOI:** 10.3389/fimmu.2023.1087595

**Published:** 2023-02-17

**Authors:** Nada Sherif Amin, Mostafa K. Abd El-Aziz, Mohamed Hamed, Ramez Reda Moustafa, Hend M. El Tayebi

**Affiliations:** ^1^Clinical Pharmacology and Pharmacogenomics Research Group, Department of Pharmacology and Toxicology, Faculty of Pharmacy and Biotechnology, German University in Cairo, Cairo, Egypt; ^2^Department of Neurology, Faculty of Medicine, Al-Azhar University, Cairo, Egypt; ^3^Department of Neurology, Faculty of Medicine, Ain Shams University, Cairo, Egypt

**Keywords:** pharmacogenomics, multiple sclerosis, autoimmune disease, disease-modification therapies, personalized medicine

## Abstract

**Background:**

Multiple sclerosis (MS) is characterized by a complex etiology that is reflected in the lack of consistently predictable treatment responses across patients of seemingly similar characteristics. Approaches to demystify the underlying predictors of aberrant treatment responses have made use of genome-wide association studies (GWAS), with imminent progress made in identifying single nucleotide polymorphisms (SNPs) associated with MS risk, disease progression, and treatment response. Ultimately, such pharmacogenomic studies aim to utilize the approach of personalized medicine to maximize patient benefit and minimize rate of disease progression.

**Objective:**

Very limited research is available around the long intergenic non-coding RNA (linc)00513, recently being reported as a novel positive regulator of the type-1 interferon (IFN) pathway, following its overexpression in the presence of two polymorphisms: rs205764 and rs547311 in the promoter region of this gene. We attempt to provide data on the prevalence of genetic variations at rs205764 and rs547311 in Egyptian MS patients, and correlate these polymorphisms with the patients’ responses to disease-modifying treatments.

**Methods:**

Genomic DNA from 144 RRMS patients was isolated and analyzed for genotypes at the positions of interest on linc00513 using RT-qPCR. Genotype groups were compared with regards to their response to treatment; additional secondary clinical parameters including the estimated disability status score (EDSS), and onset of the disease were examined in relation to these polymorphisms.

**Results:**

Polymorphisms at rs205764 were associated with a significantly higher response to fingolimod and a significantly lower response to dimethylfumarate. Moreover, the average EDSS of patients carrying polymorphisms at rs547311 was significantly higher, whereas no correlation appeared to exist with the onset of MS.

**Conclusion:**

Understanding the complex interplay of factors influencing treatment response is pivotal in MS. One of the factors contributing to a patient’s response to treatment, as well as disease disability, may be polymorphisms on non-coding genetic material, such as rs205764 and rs547311 on linc00513. Through this work, we propose that genetic polymorphisms may partially drive disease disability and inconsistent responses to treatment in MS; we also aim to draw attention towards genetic approaches, such as screening for specific polymorphisms, to possibly direct treatment choices in such a complex disease.

## 1 Introduction

Multiple sclerosis (MS) is a disorder of the central nervous system (CNS), causing neurological disabilities in young adults. This complex and multifactorial disease affects more than 2.5 million people globally, with a higher prevalence in females compared to males (1). Establishing prevalence and estimates of MS in developing countries is yet to be made more feasible, primarily due to the lack of epidemiological studies around this disease. Treatmenst options of MS are aimed at 3 disciplines; the management of acute relapses, symptomatic treatment, and disease modifying treatment (DMT) (2). DMTs are drugs that are aimed at modulating immune responses. The primary goal of using DMTs is controlling and integrating clinical parameters such as relapses or disease progression, and magnetic resonance imaging (MRI) parameters such as the presence of new lesions. Together, both parameters are combined in a term called no evidence of disease activity (NEDA) (3). Despite the availability of well-established evidence on the clinical efficacy of these drugs, inconsistent treatment responses still prevail, providing a frequently insurmountable barrier against achieving adequate clinical outcomes and providing a better quality of life for these patients.

Personalized therapies for MS are recently gaining a rightful interest, where the integration of parameters beyond MRI scans and disease state has a potential for contributing to better and more efficient treatment choices. Such parameters include accounting for differential epigenetic profiles in patients vs. healthy subjects, an emerging and promising area of research (4), as well as possibly accounting for genetic variances, or single nucleotide polymorphisms (SNPs), whose downstream effects may ultimately translate into affecting the response to treatment in patients who were typically suited for that given treatment (5). SNPs accounting for such discrepancies are not uncommon in MS. While accounting for these SNPs would certainly be pivotal in influencing the choice of DMT, a gap would still remain, since all SNPs previously associated with treatment responses were on protein coding elements (6, 7). Indeed, the insurmountable epigenetic component of MS calls for the imminent bridging between the inconsistent treatment responses and SNPs on both coding and non-coding genetic elements, integrating both the epigenetic component of the disease as well as potential implications of genetic variations.

The role of long non-coding RNAs (lncRNAs) is recently emerging in MS, owing to the high regulatory capacity of these elements in the disease pathogenesis (8–11). LncRNAs are non-coding species exceeding 200 nucleotides in length, and they can influence the differentiation of oligodendrocytes and the polarization state of macrophages, act as micro-RNA (miRNA) sponges, regulate the levels of immune-modulatory cytokines, as well as influence the activation state of CD4+ cells. It, therefore, comes as no surprise that SNPs occurring on such elements are expected to play important roles in the downstream activity of a given lncRNA, potentially extending to alterations in treatment responses among different patients.

Long intergenic non-coding RNA (linc)00513 has been recently reported as a novel regulator of the type 1 interferon (IFN) signaling pathway (12). Polymorphisms in the promotor region of linc00513 (G for rs205764 and A for rs547311) have also been associated with an overexpression of linc00513 and a subsequent increase in the downstream signaling activity of the type 1 IFN pathway (12). In MS, no such variances have yet been investigated, and a corresponding role of linc00513 remains elusive. Given the pivotal role that the type 1 IFN signaling pathway plays in MS (13–16), investigating the implications of these genetic variations in MS patients seemed of great interest. We therefore aim to provide data on the distribution of genotypes at rs205764 and rs547311 in MS patients of the Egyptian population, and correlate these genotypes with the response to treatment. Other clinical parameters are also included, further asserting the clinical ramifications of these SNPs.

## 2 Materials and methods

### 2.1 Study group

This study included 144 RRMS patients (115 females and 29 males) with a clinical diagnosis of MS. Clinical parameters of the patients were assessed by the same neurologist at Nasser Institute Hospital MS Unit, Cairo, Egypt. Information was obtained regarding the patients’ response to treatment, which was defined as the lack of clinically documented attacks for at least one year on treatment (17). Additional information on the age of onset, EDSS, and the annualized relapse rate (ARR) – a parameter reflecting the number of relapses per year, were also obtained. All patients included were older than 18 years of age, diagnosed at the same MS center, and on a given medication for one year at the time of the study. Alternatively, their medical records were retrospectively checked, when applicable, for their status at one year of treatment in order to eliminate the effect of treatment duration on response. The age of onset was defined from the time of symptom onset not from the time of diagnosis, and the EDSS and ARR were assessed and calculated by the neurologist.

First, with regards to the response to treatment, patients were considered responsive to a given medication if they experienced no relapses within the first year of treatment initiation. Alternatively, relapses occurring within the first few months of treatment were considered a positive predictor of treatment inefficacy in these particular patients (17), and they were therefore considered non-responsive to the given medication. All 144 RRMS patients were initially compared for differences in the frequency of responders among the genotype groups to highlight potential genotype-treatment response association. In subsequent subgrouping based on the treatment received, n = 48 patients receiving fingolimod, and n = 19 patients receiving DMF, were analyzed for the frequency of responders among the different genotype groups (**Tables 1**, **2**); analysis of the response to treatment was done after one year of treatment initiation. For the EDSS, the scores of n = 108 (for rs205764) and n = 110 (for rs547311) RRMS patients were compared with regards to their genotypes to highlight potential genotype-EDSS association (**Table 3**). All study participants signed an informed consent, and this study was approved from the ethics committees at the German University in Cairo and Nasser Institute Hospital, Cairo, Egypt.

**Table 1:** Difference in the response to fingolimod between patients carrying different alleles at both locations, with regards to different modes of inheritance for rs205764.

**SNP**	**Mode of Inheritance**	**Genotype**	**n**	**responsive**	**nonresponsive**	**p-value**
**rs205764**	**Dominant**	T/T	17	10	7	0.0362
		G/T + G/G	31	27	4	
	**Recessive**	G/G	4	4	0	0.5607
		G/T + T/T	44	33	11	
	**Overdominant**	G/T	32	25	7	0.1736
		G/G + T/T	23	15	8	
	**Codominant**	T/T	17	10	7	0.0672
		G/G	4	4	0	
		G/T	27	23	4	
**rs547311**	**Dominant**	G/G	17	11	6	0.103
		A/G + A/A	31	26	5	

A significant difference was found in the response to fingolimod between patients carrying one or two G alleles at rs205764 compared to those carrying two T alleles. SNP, single nucleotide polymorphism.

**Table 2:** Difference in the response to DMF between patients carrying different alleles at both locations, with regards to the different modes of inheritance for rs205764.

SNP	Mode of Inheritance	Genotype	n	responsive	nonresponsive	p-value
**rs205764**	**Dominant**	T/T	7	7	0	0.0436
		G/T + G/G	12	6	6	
	**Recessive**	G/G	2	1	1	>0.9999
		G/T + T/T	17	12	5	
	**Overdominant**	G/T	10	5	5	0.1698
		G/G + T/T	9	7	2	
	**Codominant**	T/T	7	7	0	0.0775
		G/G	2	1	1	
		G/T	10	5	5	
**rs547311**	**Dominant**	G/G	7	5	2	>0.9999
		A/G + A/A	12	8	4	

A significant difference was found in the response to DMF between patients carrying one or two G alleles at rs205764 compared to those carrying two T alleles SNP, single nucleotide polymorphism. DMF, dimethyl fumarate.

**Table 3:** Difference in the average EDSS between patients carrying different alleles at both locations, with regards to different modes of inheritance for rs547311.

SNP	Mode of Inheritance	Genotype	n	Average EDSS	p-value
**rs205764**	**Dominant**	T/T	53	2.217	0.201
		G/T + G/G	55	2.3455	
**rs547311**	**Dominant**	G/G	59	2.1186	0.0419
		A/G + A/A	51	2.402	
	**Recessive**	A/A	10	2.45	0.323
		A/G + G/G	97	2.232	
	**Overdominant**	A/G	38	2.5132	0.1698
		A/A + G/G	70	2.1429	
	**Codominant**	G/G	59	2.1186	0.1142
		A/A	10	2.45	
		A/G	37	2.432	

A significant difference was found in the average EDSS between patients carrying one or two A alleles at rs547311 compared to those carrying both G alleles.SNP, single nucleotide polymorphism; EDSS, estimated disability status score.

### 2.2 Molecular research methodology

#### 2.2.1 Genomic DNA isolation

Genomic DNA was isolated from patients’ whole blood using QiAmp DNA extraction kit (Qiagen, USA) according to manufacturer’s protocol. DNA samples were stored at -20 C until downstream processing. DNA was quantified using Nanodrop.

#### 2.2.2 Identification of the polymorphisms of interest

Genotyping experiments were performed on StepOne Real Time Quantitative PCR (RT-qPCR) (Applied Biosystems, USA), using TaqMan reagents: TaqMan Genotyping Mastermix and TaqMan SNP Assays with their corresponding *unique* Assay IDs (rs205764: C:7614549_10; rs547311: C:2595518_10) (Life Technologies, USA). Fluorescence signals detected were VIC and FAM.

Preparation of the reaction mixture:

Each PCR tube contained a volume of DNA equivalent to at least 20 ng (manufacturer’s recommendation), nuclease-free water to 11.25 ul, 12.5 ul TaqMan Genotyping Mastermix, and finally, 1.25 ul TaqMan SNP Assay.

The standard thermal profile was used (**Table 4**).

**Table 4:** Thermal profile used for amplification of genomic DNA.

	Pre-PCR Read	Thermal Cycling			Post-PCR Read	
Stage/Step	Holding Stage	Holding Stage	Cycling (50 cycles)		Holding Stage	
			Denature	Anneal/Extend		
Temperature	60°C	95°C	92°C	60°C	60°C
Time (mm:ss)	00:30	10:00	00:15	01:00	00:30
Data Collection	Yes	No	No	Yes	Yes

PCR, polymerase chain reaction.

### 2.3 Statistical analysis

Statistical analysis was performed on GraphPad Prism v9.4 using parametric/nonparametric t-test/one-way ANOVA when comparing the age of onset, EDSS, and the ARR, and using Fisher exact when comparing the response to treatment. A p-value < 0.05 was considered as statistically significant. Values on the graphs are expressed as mean ± SEM.

**Figure 1:**
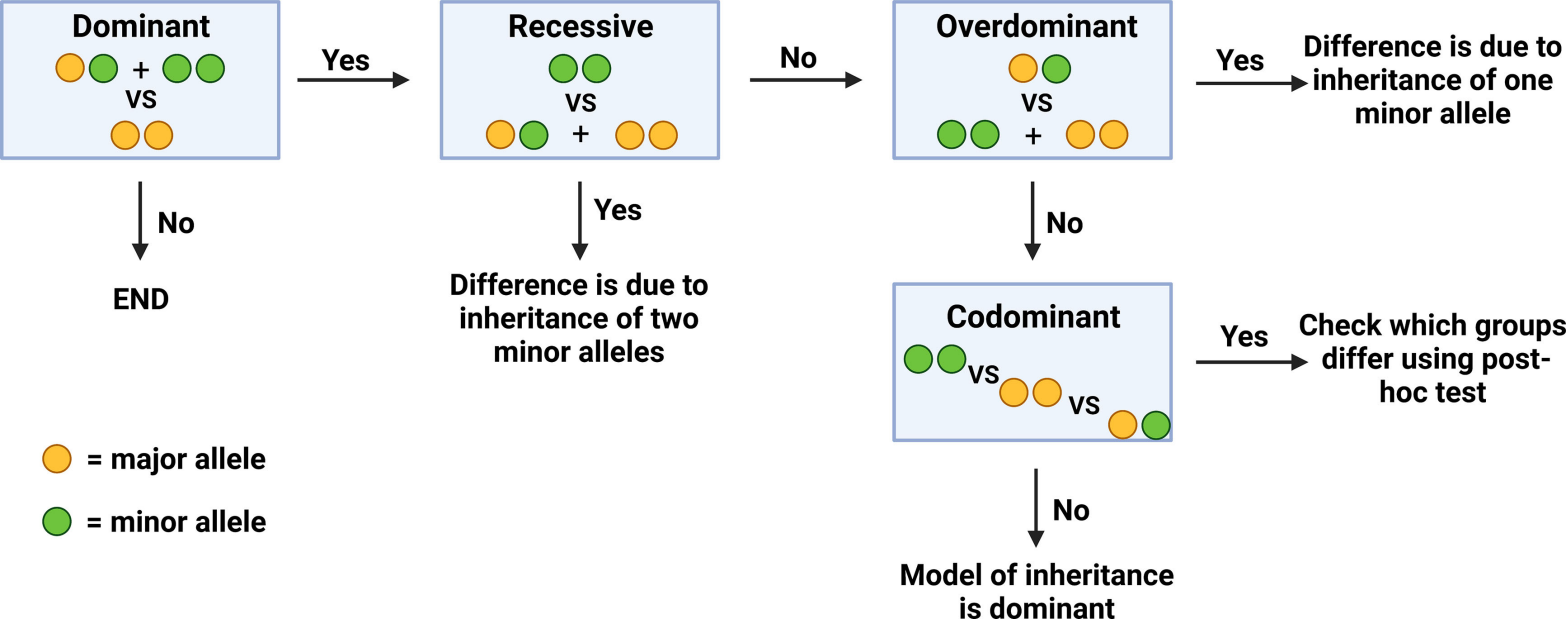
Algorithm of comparison between different genotype groups.

In order to determine the exact genotype that correlates to a significant difference in a given clinical parameter (inheritance of one VS two minor alleles), genotypes at the two polymorphisms were analyzed and compared with regards to four different models of inheritance. Initially, all samples were compared with regards to the dominant model of inheritance, where patients carrying the homozygous major genotype were compared to the rest of the patients. If significant differences were found in this model, additional models of inheritance were subsequently examined in order to identify if this difference was due to the inheritance of one or two minor alleles. In the recessive model of inheritance, patients who were homozygous for the minor allele were compared to the rest of the patients. In the overdominant model, patients who were heterozygous were compared to the rest of the patients. Finally, in the codominant model, all three genotypes were compared to each other (**Figure 1**).

## 3 Results

### 3.1 Patient characteristics

This study group consisted of 79.7% females (n=115) and 20.2% males (n=29). The EDSS and the age of onset were not gender-dependent (p>0.05). Patient characteristics are summarized in **Table 5**.

**Table 5:** Patient characteristics.

	n (%)*
**Gender**	M 29 (20.2)
	F 115 (79.7)
**MS subtype**	RRMS 144 (97.2)
**Treatment**	Gilenya 48 (32.4)
	Rebif 35 (23.6)
	Marovarex 19 (12.8)
	Avonex 15 (10.1)
	Aubagio 14 (9.45)
	Betaferon 4 (2.7)
	Other 2 (4.05)
**Age**	33 (18-62)
**Disease duration**	<5 88 (61.4) ≥5 56 (38.5)
**Onset age**	≤ 20 30 (20.9)
	Over 20 114 (79.05)
**Response to treatment**	Yes 107 (72.2)
	No 41 (27.7)

*median(range) reported for age.

M, males; F, females; MS: multiple sclerosis; RRMS, relapsing-remitting MS.

### 3.2 Genotyping results

The genotype distribution for both polymorphisms were as follows:

For rs205764, 70 were homozygous for the allele T (48.6%), 12 were homozygous for the allele G (8.3%), and 62 were heterozygous (43%). For the investigated subset of MS population, T was considered as the major allele and G was considered as the minor allele according to the genotyping results.

For rs547311, 76 were homozygous for the allele G (52.7%), 15 were homozygous for the minor A (10.4%), and 52 were heterozygous (36.11%). For the investigated subset of MS population, G was considered as the major allele and A was considered as the minor allele according to the genotyping results. Genotyping results and classification are summarized in **Table 6**.

**Table 6:** Distribution of genotypes among the rs205764 and rs547311.

SNP	Mode of Inheritance	Genotype	n	%
**rs205764**	**Dominant**	T/T	70	48.6
		G/T + G/G	74	51.3
	**Recessive**	G/G	12	8.3
		G/T + T/T	132	91.6
	**Overdominant**	G/T	62	43
		G/G + T/T	82	56.9
	**Codominant**	T/T	70	48.6
		G/G	12	8.7
		G/T	62	43
**rs547311**	**Dominant**	G/G	76	52.3
		A/G + A/A	67	46.5
	**Recessive**	A/A	15	10.4
		A/G + G/G	128	88.9
	**Overdominant**	A/G	52	36.1
		A/A + G/G	91	63.1
	**Codominant**	G/G	76	52.3
		A/A	15	10.4
		A/G	52	36.1

SNP, single nucleotide polymorphism.

### 3.3 Analyzing the response to treatment in different genotype groups for rs205764 and rs547311

The response to treatment was defined as the lack of clinically documented attacks for at least one year on treatment (17–19), as previously mentioned. No significant differences were found in the response to treatment in general, between patients carrying polymorphisms at either location and those who do not, either compared as a whole or sub-grouped by gender.

**Figure 2:**
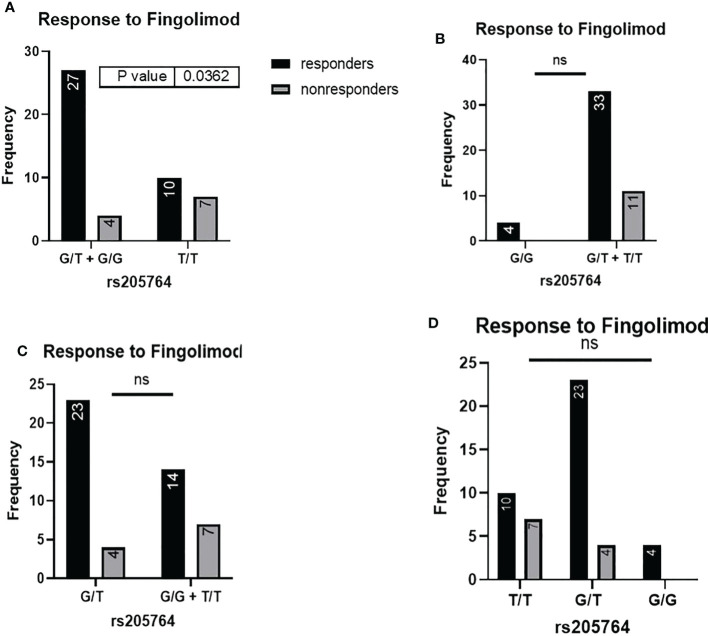
Response to fingolimod in patients carrying polymorphisms at rs205764. **(A)** a significant difference was found in the response to fingolimod between patients carrying either one or two G alleles compared to those carrying both T alleles (dominant). No significant differences were found in the other modes of inheritance, shown in **(B)** recessive, **(C)** overdominant, and **(D)** codominant.

**Figure 3:**
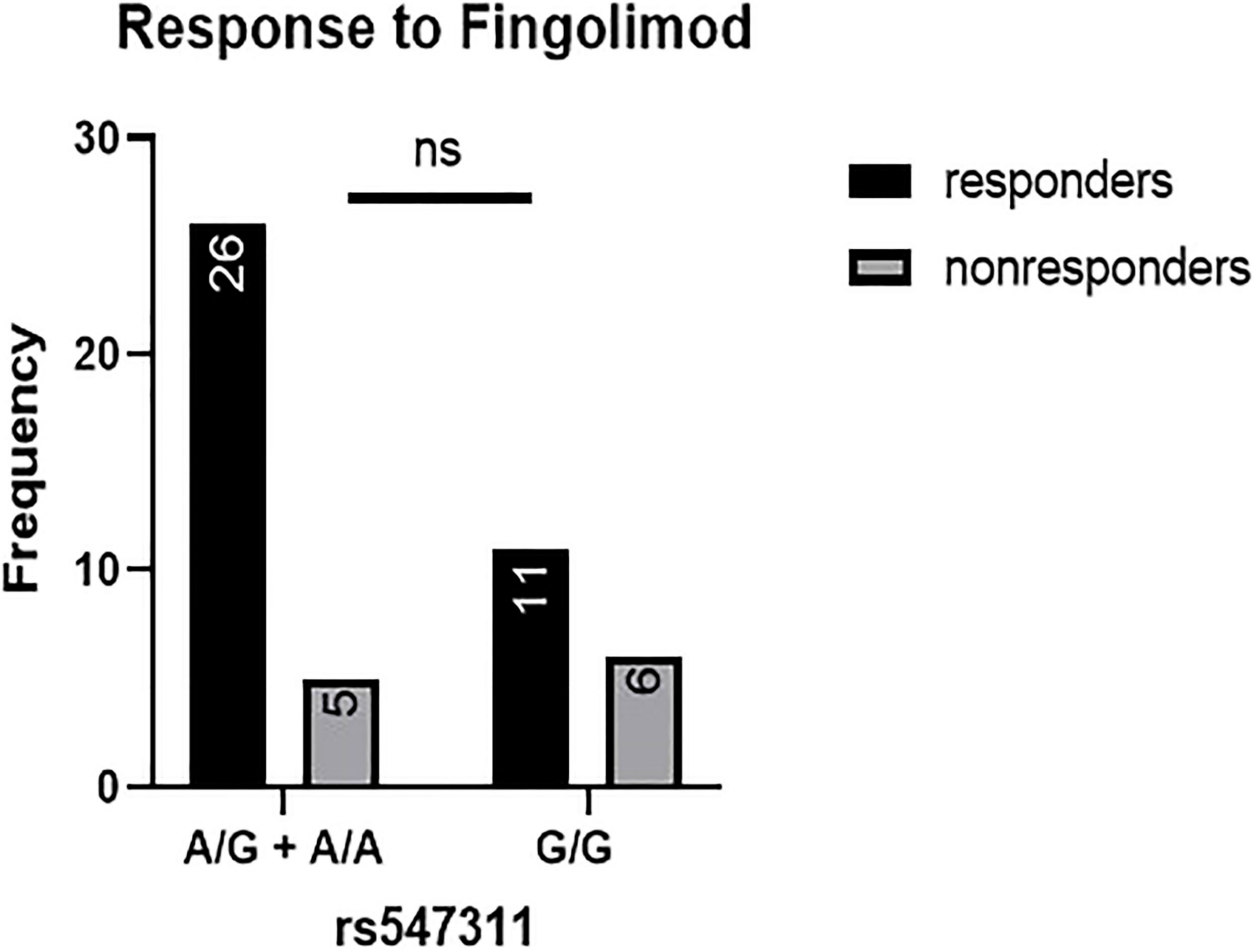
Response to fingolimod in patients carrying polymorphisms at rs547311. No significant difference was found in the response between patients carrying either one or two A alleles at rs547311 and those carrying both G alleles.

**Figure 4:**
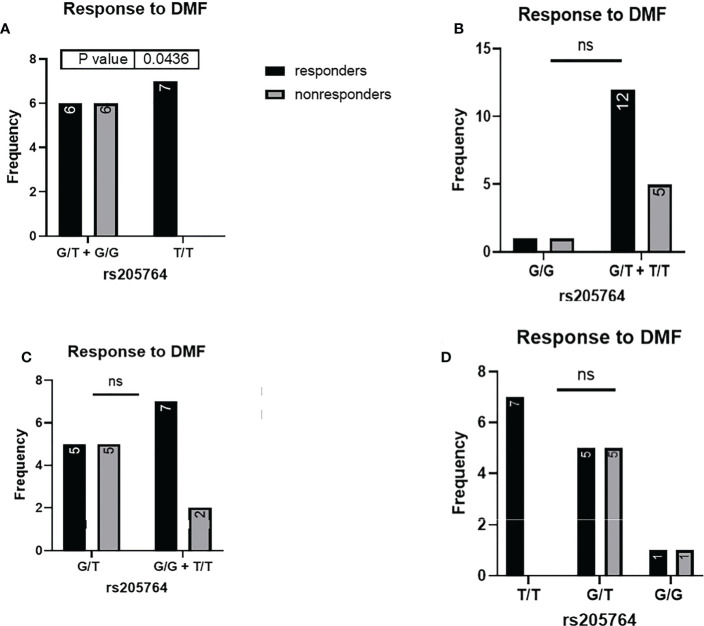
Response to DMF in patients carrying polymorphisms at rs205764. **(A)** a significant difference was found in the response to DMF between patients carrying either one or two G alleles compared to those carrying both T alleles (dominant). No significant differences were found in the other modes of inheritance, shown in **(B)** recessive, **(C)** overdominant, and **(D)** codominant. DMF: dimethyl fumarate.

**Figure 5:**
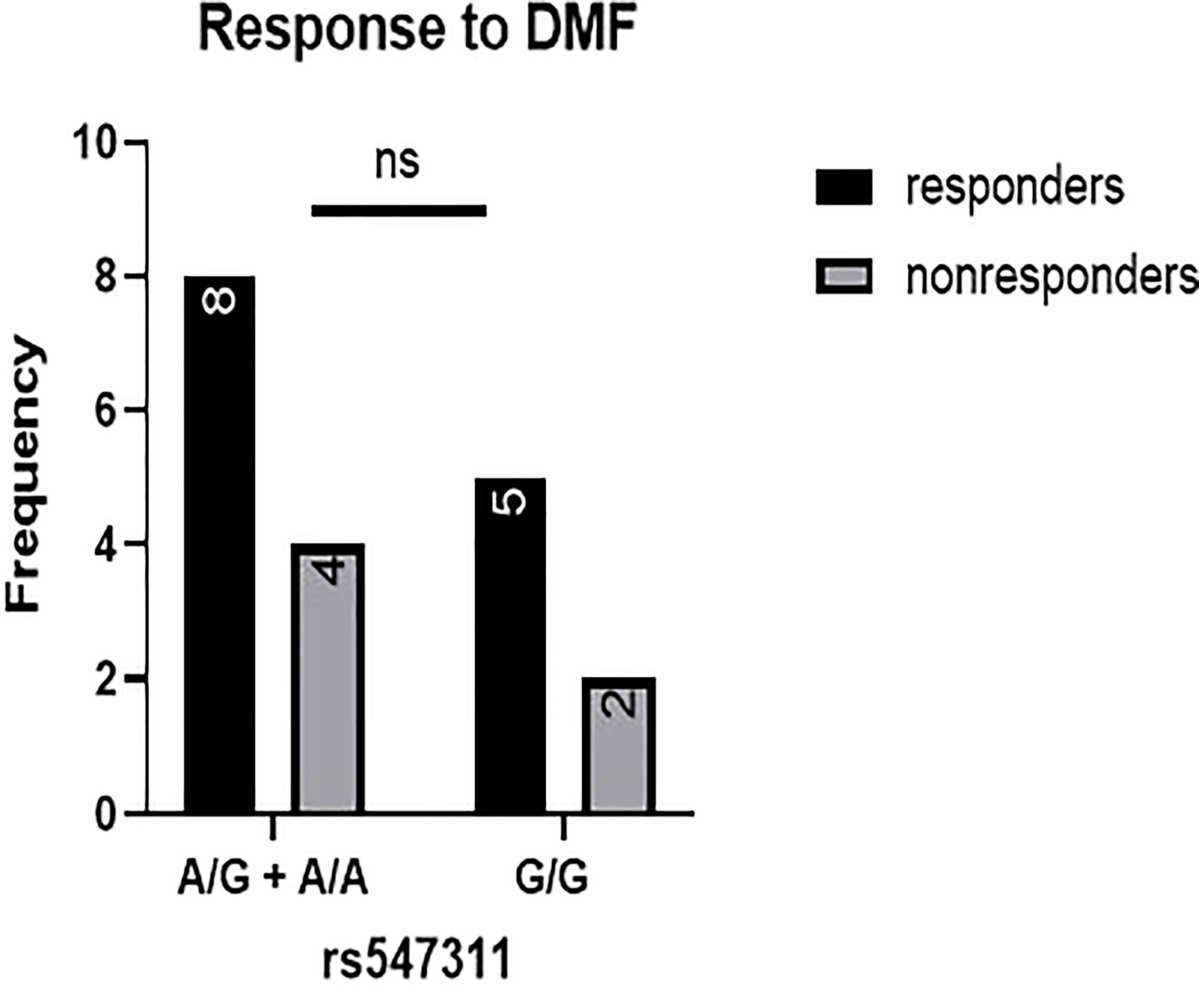
Response to DMF in patients carrying polymorphisms at rs547311. No significant difference was found in the response between patients carrying either one or two A alleles at rs547311 and those carrying both G alleles. DMF: dimethyl fumarate.

When comparing the response of patients to specific DMTs (**Figures 2**–**5**), patients carrying polymorphisms at rs205764 in the dominant model (either one or two G alleles) showed a significantly higher response to fingolimod (p = 0.0362*) with an odds ratio (OR) of 4.72 compared to patients carrying two T alleles (**Table 1**). These patients also showed a significantly lower response to DMF (p = 0.0436*) with a relative risk of 0.5 (**Table 2**). Upon comparing these patients based on other models of inheritance, starting with the recessive, followed by the overdominant and the codominant, no significant difference was observed, suggesting that the difference in responses to fingolimod or DMF could equally be attributed to inheritance of either one or two G alleles at rs205764. Patients carrying polymorphisms at rs547311 showed no statistically significant differences in their response to fingolimod (p = 0.103), or DMF (p > 0.999).

### 3.4 Analyzing other clinical parameters in different genotype groups for rs205764 and rs547311

#### 3.4.1 EDSS

**Figure 6:**
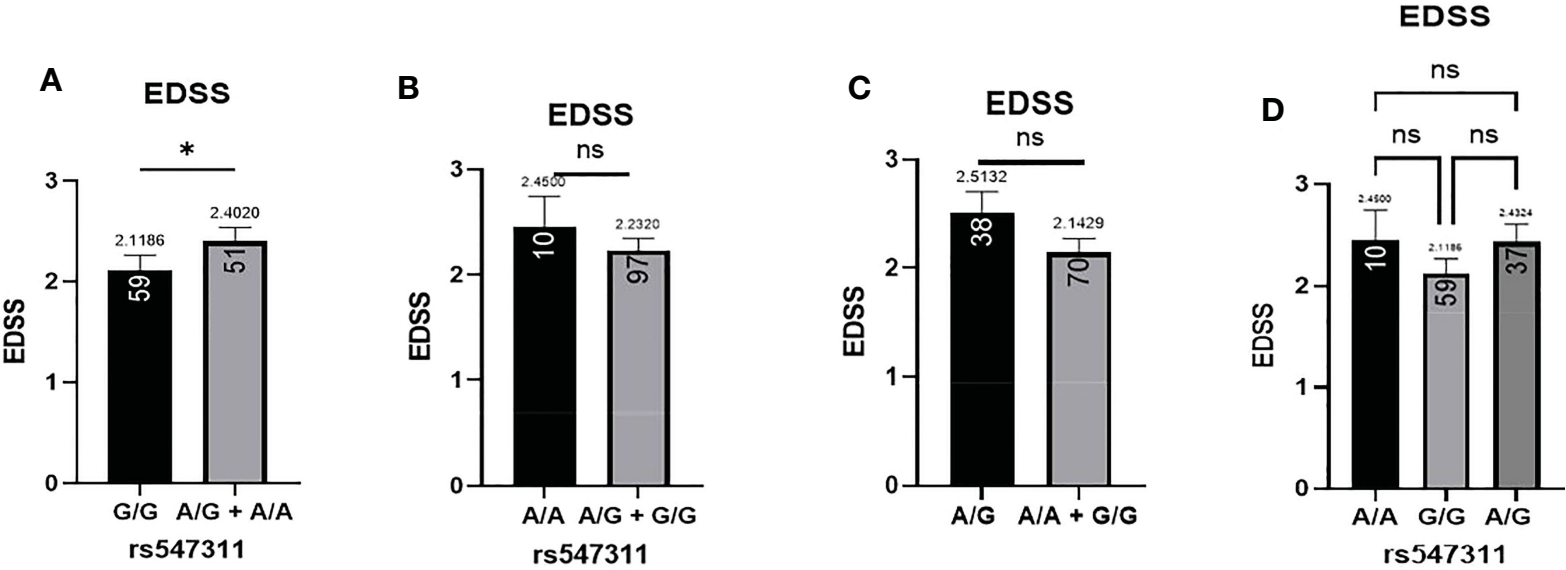
Difference in the average EDSS between patients carrying polymorphisms at rs547311. **(A)** a significant difference was found in the EDSS between patients carrying either one or two A alleles compared to those carrying both G alleles (dominant). No significant differences were found in the other modes of inheritance, shown in **(B)** recessive, **(C)** overdominant, and **(D)** codominant. DMF, dimethyl fumarate. EDSS, estimated disability status score.

**Figure 7:**
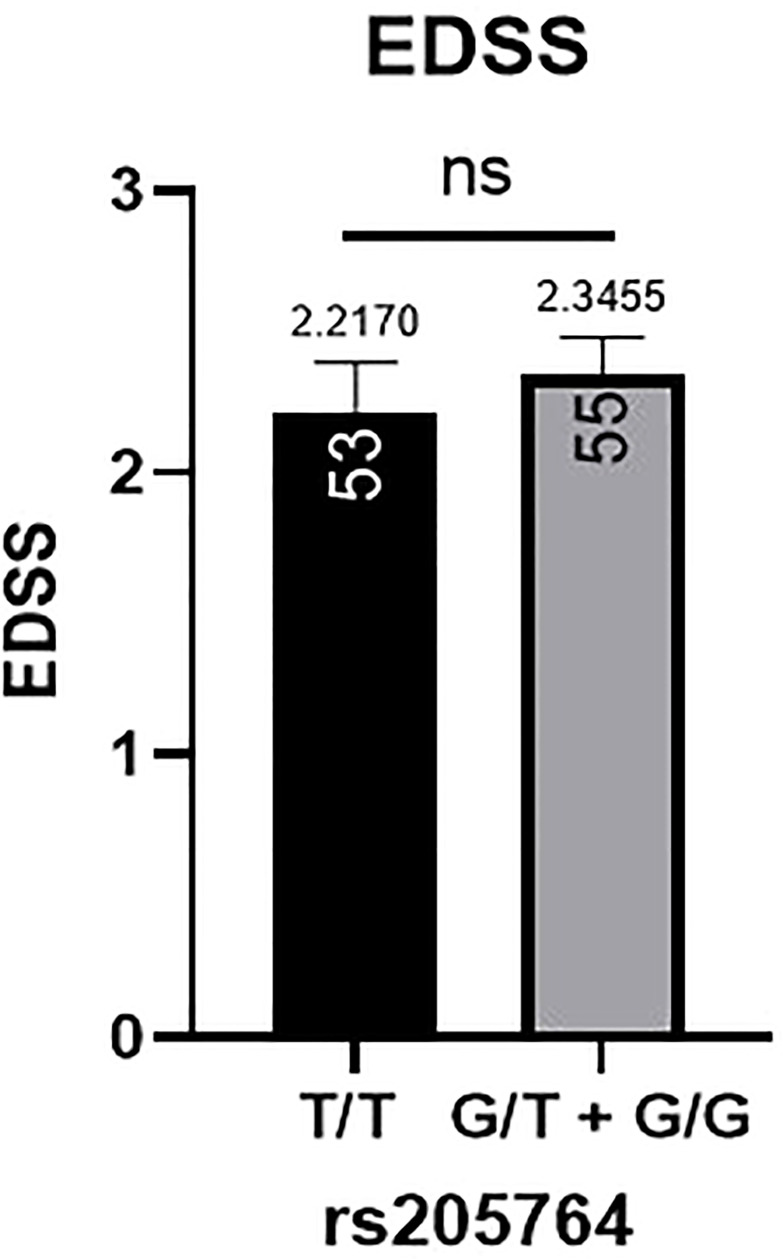
Difference in the average EDSS between patients carrying polymorphisms at rs205764. No significant difference was found in the average EDSS between patients carrying either one or two G alleles compared to those carrying both T alleles. EDSS, estimated disability status score.

Patients’ EDSS were assessed by the same consulting neurologist at Nasser Institute Hospital. When comparing the average EDSS of patients carrying different alleles at both positions (**Figures 6**, **7**), patients carrying one or two A alleles at rs547311 showed a significantly higher EDSS (p = 0.0419*) compared to patients carrying two G alleles. Upon comparing these patients based on other models of inheritance, no significant difference was observed, suggesting, again, that the inheritance of one or two A alleles at rs547311 may be equally detrimental for a patient’s EDSS. Different alleles at rs205764, on the other hand, showed no significant association with the patients’ EDSS (**Table 3**).

#### 3.4.2 Age of onset

**Figure 8:**
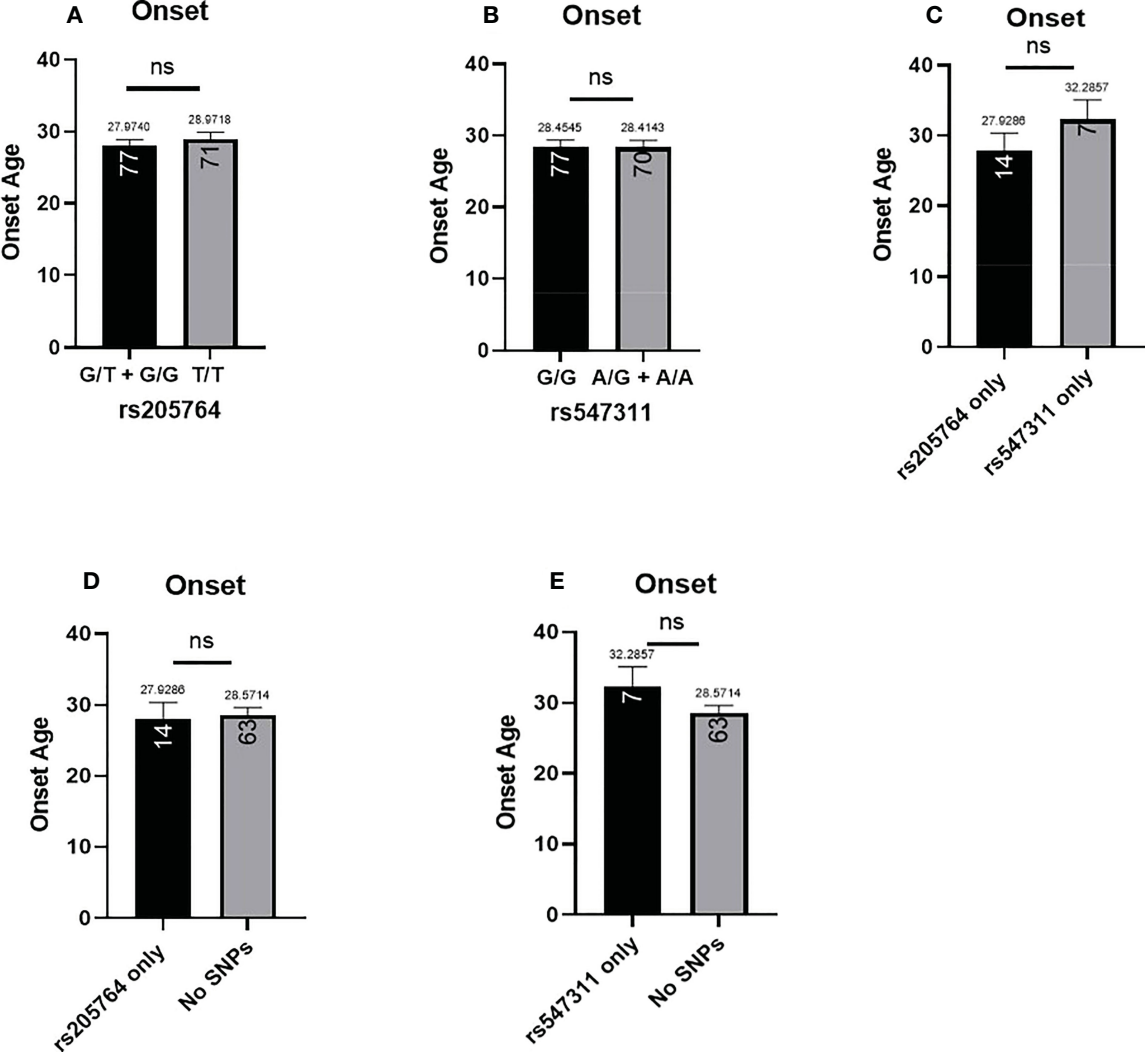
Difference in the average age of onset between patients carrying polymorphisms at either rs205764 or rs547311. No significant difference was found in the age of onset between patients carrying one or two G alleles at rs205764 and those carrying two T alleles **(A)**. For rs547311, no significant difference was found between patients carrying one or two A alleles compared to those carrying two G alleles (dominant) **(B)**. The same was true upon comparing patients carrying either polymorphism exclusively without the other **(C)**, as well as when comparing patients carrying either polymorphism to those carrying two major alleles **(D, E)**.

The patients’ age of onset was defined from the reported time of onset of symptoms and not the time of diagnosis. The average age of onset of different patient genotype groups were compared for the two polymorphic locations (**Figure 8**). When comparing the average age of onset between patients carrying one or two G alleles at rs205764, no significant difference was observed (p = 0.7098). This was also the case when comparing patients carrying polymorphisms at rs205764 only (i.e. carrying the major G allele at rs547311) (p = 0.8934). The opposite was also true for rs547311. Additionally, when comparing the average age of onset for patients carrying a polymorphism at either location exclusively without the other (**Figure 8C**), a trend could be seen, yet the difference did not reach significance (p = 0.3683). These results are summarized in **Table 7**.

**Table 7:** Difference in the average age of onset and the ARR between patients carrying different alleles at both locations.

SNP	Mode of Inheritance	Genotype	n	Average Onset Age (years)	p-value
**rs205764**	**Dominant**	T/T	71	28.97	0.7098
		G/T + G/G	77	27.97	
**rs547311**	**Dominant**	G/G	77	28.45	0.8289
		A/G + A/A	70	28.41	
**rs205764 only**	**Dominant**	T/T + No SNP2	63	28.57	0.8934
		G/T + G/G	14	27.92	
**rs547311 only**	**Dominant**	G/G + No SNP1	63	28.57	0.2597
		A/G + A/A	7	32.85	
**Exclusive polymorphisms**	**Dominant**	G/T + G/G	14	27.92	0.3683
		A/G + A/A	7	32.85	
**ARR**				**Average ARR**	
**rs205764**	**Dominant**	T/T	62	1.32	0.7815
		G/T + G/G	73	1.29	
**rs547311**	**Dominant**	G/G	70	1.27	0.6016
		A/G + A/A	61	1.34	
**rs205764** **(fingolimod)**	**Dominant**	T/T	16	1.28	0.650
		G/T + G/G	27	1.39	
**rs547311** **(fingolimod)**	**Dominant**	G/G	18	1.25	0.475
		A/G + A/A	25	1.43	
**rs205764** **(DMF)**	**Dominant**	T/T	6	1.05	0.386
		G/T + G/G	12	1.3	
**rs547311** **(DMF)**	**Dominant**	G/G	8	1.29	0.621
		A/G + A/A	9	1.14	

ARR, annualized relapse rate.

#### 3.4.3 ARR

The patients’ ARR was calculated by the neurologist and compared across the different genotypes in the dominant model with regards to the two polymorphisms. Although non-responders, by definition, experience more relapses than responders, and should be expected to have a higher ARR, no significant difference in the ARR between genotypes at either location was found (**Table 7**). This is likely attributed to the fact that with the exception of fingolimod and DMF, there were no significant differences among the different genotypes with regards to the response to MS treatment in this study. However, upon comparing the ARR between genotypes *within* a given treatment (for both fingolimod and DMF – **Table 7**), the lack of significant differences persists, presenting the usefulness of assessing treatment responses in terms of more than one analysis in this study. Moreover, the effect of patient genotype on ARR may be better assessed through measuring differential changes in ARR before and after treatment for each genotype.

### 3.5 Correlation between age and the analyzed parameters

In order to ascertain that the analyzed parameters are not influenced by age in our studied patient cohort, a correlation was done between age and each of the EDSS, response to fingolimod, response to DMF, as well as the ARR. Correlations between age and each of EDSS and ARR was done using Pearson correlation, and with the response to treatment using Point-Biserial correlation test. None of the correlations with age appeared to be major nor significant, suggesting that in our cohort, age did not influence any of these parameters. These results are summarized in **Table 8**.

**Table 8:** Correlation between age and the analyzed clinical parameters.

	EDSS	Response to fingolimod	Response to DMF	ARR
**R**	0.1581	0.1818	0.309	0.0979
**R^2^**	0.024	0.033	0.095	0.0096
**p-value**	0.102	0.231	0.197	0.264

EDSS, estimated disability status score; DMF, dimethyl fumarate; ARR, annualized relapse rate.

## 4 Discussion

Multiple sclerosis is a complex, multifactorial, immune-mediated disease targeting the CNS, causing focal lesions of demyelination, impairing nerve conduction and signal transmission (1). Treatment strategies of the disease are generally aimed at 3 directions, of particular controversy and importance is the use of drugs that help modulate immune responses, called DMTs, a few examples of which are IFN-β, fingolimod, glatiramer acetate, and dimethyl fumarate (2).

Epigenetic research has garnered rightful interest in its contribution to understanding disease pathology (20), susceptibility, and development (20). Several areas of research have recently taken interest in the roles of lncRNAs in immune-mediated diseases in general, and MS in particular, in light of the pre-established epigenetic changes that are observed in the disease pathology (8–11). LncRNAs have numerous well-established genetic and epigenetic regulatory roles. Of particular interest in this frame of work is linc00513, since its dysregulation has yet been investigated in a single study conducted on systemic lupus erythematosus (SLE) patients and none is yet known about its functional role in MS. Its overexpression has been shown to positively relate to the activity of the type-1 IFN signaling pathway, contributing to the inflammatory state in SLE patients (12). Linc00513 has been identified as a risk allele for SLE in the aforementioned study, yet no such correlation has been made with MS as per the most recent MS genetic map (21). However, due to the previously well-established protective role of the same signaling pathway in MS, drawing a straightforward prediction regarding the population under investigation was not entirely possible, making it all the more intriguing to investigate its correlation to MS disease.

Single nucleotide polymorphisms (SNP)s are genetic variations involving a single base-pair. Ample research is available on SNPs involved in the development of MS; however, very little amount of research has yet taken interest in SNPs located on non-coding genetic elements, and the potential influence this may have on downstream regulatory processes, and ultimately the clinical picture of the patients. Regarding linc00513, a study has shown that G allele at rs205764 and A allele at rs547311, located in its promotor region, positively correlate to its expression levels and the subsequent signaling activity of the type-1 IFN pathway (12).

Taking it from there, the aim of this work was to determine the genetic prevalence of rs205764 and rs547311 in MS patients of the Egyptian population, and correlate these genetic variances to several clinical parameters, the primary focus of which was the response to treatment.

Blood samples were collected from 144 patients, from which genomic DNA was isolated and analyzed for the genotypes at the positions of interest on linc00513 using RT-qPCR. These polymorphisms were then correlated with the previously obtained clinical parameters of each patient: the response to treatment, onset age, and EDSS. The genotypes were analyzed and compared with regards to 4 different models of inheritance: dominant, recessive, overdominant, and codominant.

When analyzing the relationship between these polymorphisms and the patient’s response to treatment, a significant difference was found regarding patients carrying polymorphisms at rs205764, where they showed a significantly higher response to fingolimod compared to patients carrying the major allele, with an OR of 4.7. These patients also showed a significantly lower response to DMF, with an OR of 0.5. When examining additional models of inheritance, no significant differences were found, suggesting that inheritance of either one or two G alleles is equally associated with a difference in the treatment response. For the same variants, there are no reported associations, to date, with the response to treatment in MS or any other autoimmune disease. However, other variants have been studied in the context of response to DMF and fingolimod. Rs6919626, in NADPH oxidase-3 gene, has been significantly associated with a lower response to DMF, but no significant associations have been found with the response to fingolimod yet (22).

For the two remaining clinical parameters, no significant difference was seen in the average age of onset between patients carrying either polymorphism and those who do not. These variants have also not yet been previously associated with the age of onset of MS or any other autoimmune diseases; however, rs10492503 in Glypican-5 gene has previously been significantly associated with an earlier age of onset in male MS patients (23). In our study, patients carrying polymorphisms at rs547311 showed a significantly higher disability score compared to patients carrying the major allele. No significant differences were seen in the other models of inheritance, suggesting that a single or double A alleles are equally detrimental for a patient’s EDSS. Finally, polymorphisms at rs205764 appear to have no association with the EDSS. These finding appear to be partially consistent, in terms of patient disability, with the study reporting rs205764 and rs547311 as novel regulators of IFN signaling (12), where the resulting overexpression of linc00513 has been associated with a higher IFN score for SLE patients. Moreover, several other variants have previously been associated with differences in EDSS for MS patients, including rs17445836 in interferon regulatory factor-8 gene (23), rs3087456 and rs4774 in class-II trans-activator gene (24), rs1049269 in transferrin gene (25), and rs1494555 in interleukin-7 receptor gene (26).

Through this work, we intended to assert the relevance of genetic polymorphisms in the clinical course of a complex disease like MS. However, some limitations that ought to be acknowledged in this study include the small number of patients in some of the comparisons, and the lack of available data when it comes to certain clinical parameters; this includes MRI data, in which case, hindering better monitoring of the disease clinical course as well as accounting for sub-clinical disease activity, as well as patient ARR *before* treatment initiation, which would have been substantially beneficial in assessing the differential treatment efficacies among the different genotypes from a relapse-incidence perspective, further corroborating the significant differences between the number of responders and non-responders found in some of the treatment groups.

The allocation of the correct patients to the correct treatment regimens is the ultimate goal in the context of any healthcare specialization. The development of tools, however preliminary, that aid in accomplishing this goal should be regarded with utmost priority. Establishing reliable biomarkers or screening methods for treatment stratification of MS patients is the first stepping stone towards achieving truly personalized MS therapy. This could potentially be achieved through exploring the possibility of constructing a gene panel consisting of all SNPs that are implicated in the inconsistent treatment responses among MS patients, and potentially using it as a guide to direct physicians towards more effective treatment choices, maximizing patient benefits and minimizing the exposure to unnecessary therapies, and possibly untying one of the knots contributing to the complexity of this multifactorial disease.

## Data availability statement

The original contributions presented in the study are included in the article/supplementary materials. Further inquiries can be directed to the corresponding author.

## Ethics statement

The studies involving human participants were reviewed and approved by German university in cairo ethics committee and Nasser Institute hospital ethics committee. The patients/participants provided their written informed consent to participate in this study.

## Author contributions

HE designed the research framework and methodology. NA carried out sample collection, DNA isolation and genotyping, statistical analysis and manuscript writing. ME-A contributed to sample collection and DNA isolation. RR and MH were the neurologists who provided crucial clinical data including EDSS among other parameters, and also assisted in the ethical approval of this study. All authors contributed to the article and approved the submitted version.
